# Evaluating antimalarial efficacy in single-armed and comparative drug trials using competing risk survival analysis: a simulation study

**DOI:** 10.1186/s12874-019-0748-2

**Published:** 2019-05-17

**Authors:** Prabin Dahal, Philippe J. Guerin, Ric N. Price, Julie A. Simpson, Kasia Stepniewska

**Affiliations:** 1 WorldWide Antimalarial Resistance Network (WWARN), Oxford, UK; 2 Centre for Tropical Medicine and Global Health, Nuffield Department of Clinical Medicine, University of Oxford, Oxford, UK; 3 Global and Tropical Health Division, Menzies School of Health Research and Charles Darwin University, Darwin, Australia; 4 Centre for Epidemiology and Biostatistics, Melbourne School of Population and Global Health, The University of Melbourne, Melbourne, Australia

**Keywords:** Malaria, *Plasmodium falciparum*, Efficacy, Competing risk events, Cumulative incidence function

## Abstract

**Background:**

Antimalarial efficacy studies in patients with uncomplicated *Plasmodium falciparum* are confounded by a new infection (a competing risk event) since this event can potentially preclude a recrudescent event (primary endpoint of interest). The current WHO guidelines recommend censoring competing risk events when deriving antimalarial efficacy. We investigated the impact of considering a new infection as a competing risk event on the estimation of antimalarial efficacy in single-armed and comparative drug trials using two simulation studies.

**Methods:**

The first simulation study explored differences in the estimates of treatment failure for areas of varying transmission intensities using the complement of the Kaplan-Meier (K-M) estimate and the Cumulative Incidence Function (CIF). The second simulation study extended this to a comparative drug efficacy trial for comparing the K-M curves using the log-rank test, and Gray’s *k*-sample test for comparing the equality of CIFs.

**Results:**

The complement of the K-M approach produced larger estimates of cumulative treatment failure compared to the CIF method; the magnitude of which was correlated with the observed proportion of new infection and recrudescence. When the drug efficacy was 90%, the absolute overestimation in failure was 0.3% in areas of low transmission rising to 3.1% in the high transmission settings. In a scenario which is most likely to be observed in a comparative trial of antimalarials, where a new drug regimen is associated with an increased (or decreased) rate of recrudescences and new infections compared to an existing drug, the log-rank test was found to be more powerful to detect treatment differences compared to the Gray’s *k*-sample test.

**Conclusions:**

The CIF approach should be considered for deriving estimates of antimalarial efficacy, in high transmission areas or for failing drugs. For comparative studies of antimalarial treatments, researchers need to select the statistical test that is best suited to whether the rate or cumulative risk of recrudescence is the outcome of interest, and consider the potential differing prophylactic periods of the antimalarials being compared.

**Electronic supplementary material:**

The online version of this article (10.1186/s12874-019-0748-2) contains supplementary material, which is available to authorized users.

## Background

The primary endpoint in clinical studies of uncomplicated *Plasmodium falciparum* malaria is the occurrence of recrudescent parasitaemia, defined as recurrence due to the same parasite which caused the original infection. Parasite recurrence due to a heterologous parasite, which can either be a new infection with *P. falciparum* or another species of Plasmodia can potentially preclude the occurrence of recrudescence and constitute a competing risk event [1, 2]. Such scenario can occur when the parasite load of a newly acquired infection (regardless of the species or strain) outnumbers and outcompetes the low level of parasitaemia of an existing infection. A recrudescence can also be precluded when the new infection is due to a more resistant parasite strain compared to the existing susceptible parasite. These scenarios further depend on the inoculum density and the multiplication rates (efficiency) of the newly emergent infection and of the existing recrudescent parasites.

Despite advancement in statistical methods for analysing time to event outcomes [1–7], competing risk events are often ignored in the medical literature. Recent reviews have pointed out that a vast majority of studies published in high impact medical journal are susceptible to competing risk biases [8–10], and malaria is no exception. The Kaplan-Meier (K-M) survival analysis (${\widehat{S}}_{KM}(t)$) is currently recommended by the World Health Organization (WHO) for deriving antimalarial efficacy [11, 12]. Commonly the complement of the K-M estimate (${\widehat{F}}_{KM}(t)=1-{\widehat{S}}_{KM}(t)$) is reported as the WHO recommends replacing a first-line treatment with an alternative regimen if the derived estimate of cumulative failure exceeds 10% [12].

The complement of the K-M estimate provides an estimate of the marginal risk (of recrudescence), i.e. the risk of recrudescence where new infections do not occur. However, this is only possible when all enrolled participants are admitted to a hospital setting where it is not possible to get another mosquito bite, and thus, new infection. In practice, antimalarial trials are almost invariably conducted in endemic settings where new infections occur frequently and can be observed in as high as 50% of the cases [13]. The Cumulative Incidence Function (CIF) estimator proposed by Kalbfleisch and Prentice provides an alternative approach to estimate the cumulative failure by accounting for such competing risk events [14]. Several studies have compared the cumulative failure estimates derived by the complement of K-M method against the CIF estimator and have reported that the K-M approach leads to an overestimation of cumulative failure in the presence of competing risk events [9, 15–18].

The presence of competing risk events have further implications in comparative studies. Comparative antimalarial studies utilise the log-rank test for comparing the efficacy of two drugs. The log-rank test is essentially the comparison of the underlying cause-specific hazard rate between two groups [19] (see Additional file 1, Section 1 for definitions). In the absence of competing risk events, there is a one-to-one correspondence between the cause-specific hazard rate and the cumulative risk. This means that any inference drawn upon the hazard function holds equivalently true for the survival function and the cumulative risk. However, in the presence of competing risk events, this one-to-one relationship no longer holds true [20]. In such a scenario, inferences drawn using the log-rank test for comparing the equality of cause-specific hazard rates may not be valid when the interest is in comparing the cumulative risk of failure at time *t*. An alternative approach, which compares the difference in cumulative risks between two groups accounting for competing risk events, is the Gray’s *k*-sample test [21]. This is the usual log-rank test where the cause-specific hazard function is replaced by the hazard of the sub-distribution [22].

To date, there has been no comprehensive investigation of how new infections impact the analysis and interpretation of efficacy data in antimalarial trials of uncomplicated *P. falciparum* malaria. This simulation study aimed to address this gap and there were two specific objectives:

I. To quantify the magnitude of overestimation in cumulative risk of treatment failure derived by the complement of the Kaplan-Meier approach compared to the Cumulative Incidence Function in a single-armed antimalarial trial, and
II. To quantify the influence of new infections on the comparative efficacy between antimalarial drugs, by comparing two statistical tests, the log-rank test and Gray’s *k*-sample test

## Methods

Two simulation studies were carried out to explore the utility of competing risk survival analysis in single armed and comparative antimalarial drug trials. The generation of survival data is common to both of these studies and is described first.

### Generation of survival data

**Fig. 1:**
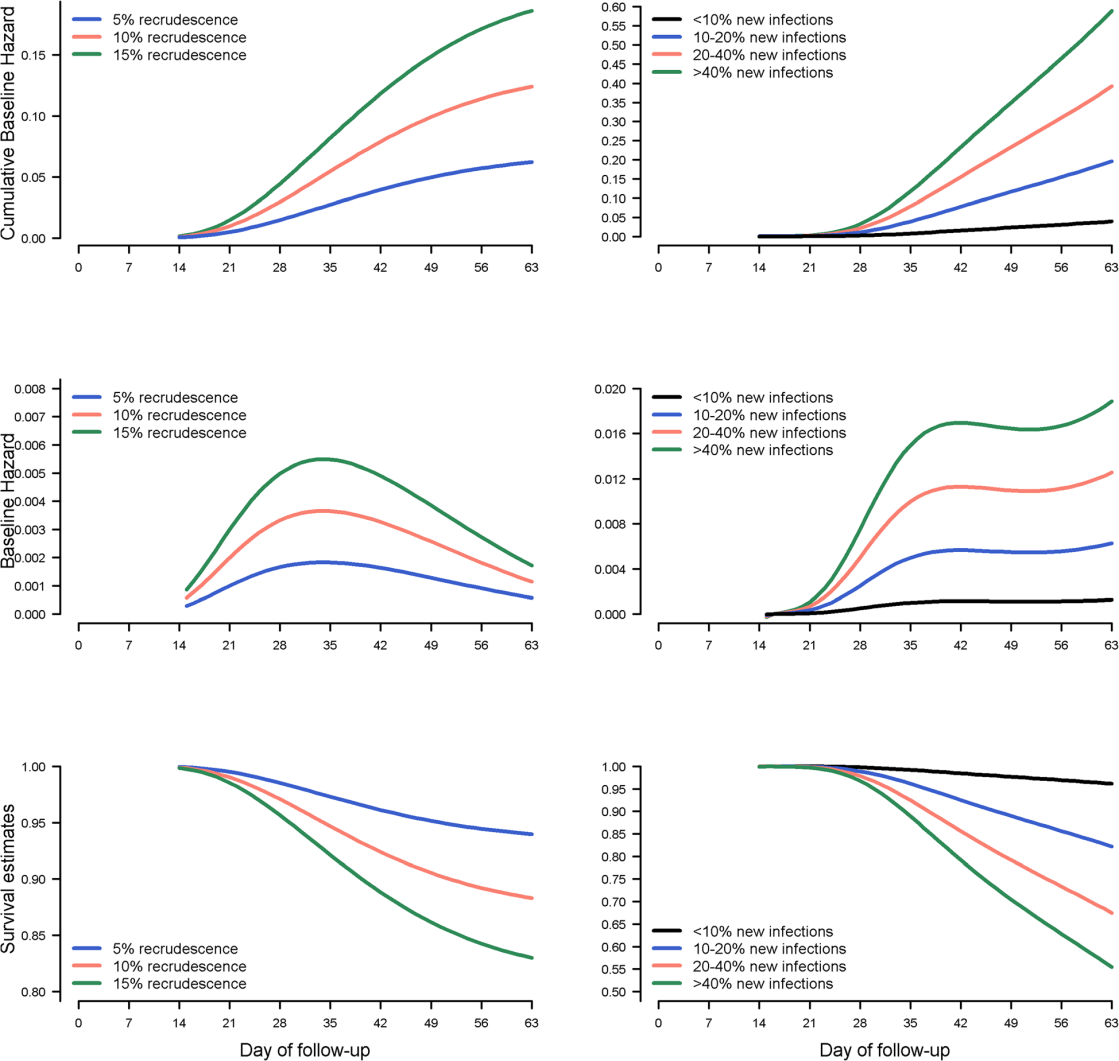
The instantaneous hazard, cumulative hazard and survival function used in simulation study I. Cumulative baseline hazard for recrudescence and new infection (top panel), respective baseline hazard function (middle panel) and survival function (bottom panel) used for generating time to recrudescence and new infection for simulation Study-I. The middle panel is the numerical derivative of the equation used for the top panel. Note that y-axes are on different scales for each plot

The time to parasitic recurrences were simulated from baseline hazard functions reflective of underlying biological mechanism of recrudescence and new infection (Fig. 1). The hazard functions were derived from individual patient outcome data from 15 studies with 4122 children aged less than 5 years for the antimalarial regimen dihydroartemisinin-piperaquine (DP). The existing studies analysed had an average efficacy of 95% in a sensitive parasite population. Fractional polynomials were used to capture non-monotonous relationship between the log of the cumulative instantaneous hazard and time to recrudescence (new infection) in order to generate survival data (manuscript currently under preparation). We then varied the intercept parameters in these two functions to explore specific scenarios outlined in the simulation studies I and II. The following cumulative baseline hazard (CBH) functions (on log scale) were used for the generation of time to recrudescence (rc), and time to new infection (ni), respectively:

1 $$ \ln \left( CBH{(t)}_{rc}\right)={\beta}_0-63.6284\times \left\{\ln {(t)}^{-1}-0.2849\right\}-0.3800\times \left\{\ln {(t)}^2-12.3188\right\} $$

2 $$ \ln \left( CBH{(t)}_{ni}\right)={\alpha}_0+9501.2150\times \left\{\ln {(t)}^{-2}-0.0858\right\}-31651.33\times \Big\{\ln {(t)}^{-2}\times \ln \left(\ln (t)-0.1054\right\}+29340.83\times \left\{{lnt}^{-2}\times \ln {(lnt)}^2-0.1294\right\}-12690.51\times \left\{\ln {(t)}^{-2}\times \ln {\left(\ln (t)\right)}^3-0.1588\right\} $$

The parameters *β*_0_ and *α*_0_ represent the intercept and were varied to achieve the desired proportion of recrudescence and new infection.

### Simulation study I: aim, design and setting

The first simulation study aimed at quantifying the magnitude of overestimation in cumulative risk of treatment failure derived by the complement of the Kaplan-Meier method compared to the Cumulative Incidence Function in a single-armed antimalarial trial.

The following combination of parasitic recurrences were generated: recrudescent proportion (5, 10, and 15%) and new infection proportion (< 10%, 10–20%, 20–40% and > 40%). The base case simulation of 5% recrudescence represents the scenario of high efficacy currently observed with the artemisinin combination therapies in Africa [23–25]. The scenarios of 10 and 15% recrudescence represent the situations likely to be observed when antimalarial drug resistance worsens, which has now been observed for some antimalarials in Cambodia and Vietnam [26–28]. New infection proportions of < 10%, 10–20%, 20–40% and > 40% progressively represent areas of very low, low, moderate and high malaria transmission settings. Standard sample size calculations are not relevant for the methodological comparisons as the aim was to compare the derived estimates of cumulative risk of treatment failure from the two methods. Trials of sample size 100, 200, 500 and 1000 patients were simulated. Sample sizes of 100 and 200 were chosen to reflect the scenarios frequently observed in antimalarial studies.

The following steps describe the simulation protocol:

i. Simulate time to recrudescence (*t*_1_) using eq. (1). The parameter *β*_0_ was varied to achieve the desired proportion of recrudescence:

- *β*_0_ =  − 3.7092 for approximately 5% recrudescence by day 63 (**base case** scenario for recrudescence)
- *β*_0_ =  − 3.0160 for approximately 10% recrudescence by day 63
- *β*_0_ =  − 2.6105 for approximately 15% recrudescence by day 63

ii. Simulate time to new infections (*t*_2_) using eq. (2). The parameter *α*_0_ was varied in order to achieve the desired proportion of new infections:

- *α*_0_ =  − 5.6004 for approximately < 10% new infection by day 63
- *α*_0_ =  − 3.9909 for approximately 10–20% new infection by day 63
- *α*_0_ =  − 3.2978 for approximately 20–40% new infection by day 63
- *α*_0_ =  − 2.8924 for approximately > 40% new infection by day 63

iii. Since early recurrences are very unlikely in patients with adequate drug exposure [25, 29], the minimum time was set to day 14 and administrative censoring was applied on the last scheduled follow-up visit (day 63). For simplicity, no losses to follow-up were assumed.
iv. For each individual, the observed time (*t*) was defined as the minimum of the simulated time to recrudescence (*t*_1_) and new infection (*t*_2_).

$$ t=\min \left({t}_1,{t}_2\right) $$

v. The final observed time was rounded to the nearest weekly visit day (7, 14, 21 and so on), reflective of the antimalarial follow-up design. The observed event corresponded to the event with minimum time, *t*, else administrative censoring was applied on day 63.
vi. For each simulated dataset, the cumulative probability of failure was estimated on days 28, 42 and 63 using the 1 minus K-M method and the CIF. New infections were censored on the day of occurrence in the 1-K-M analysis and were kept as a separate category of competing risk event when estimating the CIF.
vii. The absolute and relative differences in the two estimators derived in step (vi) were calculated.
viii. For each scenario, steps (i)-(vii) were repeated 1000 times using an acceptance sampling procedure where only datasets fulfilling the study criteria were kept (e.g. 5% recrudescence, < 10% new infection). Studies where 4–6%, 9–11% and 14–16% of recrudescences were observed were defined to have 5, 10 and 15% recrudescence, respectively. In order to achieve the desired proportion of recrudescences (approximately 5, 10 and 15%), this required a large number of simulation runs, and the first 1000 datasets fulfilling the criteria were kept for analysis.

### Simulation study II: aim, design and setting

The second simulation study aimed to quantify the influence of new infections on the comparative efficacy between antimalarial drugs, by comparing two statistical tests, the log-rank test and Gray’s *k-*sample test.

Let drug A be the current first line treatment and drug B be a new antimalarial drug under investigation. The interest is in establishing whether drug A and B are different in terms of their effect on recrudescence. The aim of the simulation was to present the results from the log-rank test for comparing the equality of the K-M curves of drug efficacies and Gray’s *k*-sample test for comparing the cumulative risks of recrudescence for drug A and drug B at day 63. For the log-rank test, new infections were censored on the time of recurrence.

Let ${\lambda}_1^A(t)$ be the cause-specific hazard function of recrudescence for drug A and ${\lambda}_2^B(t)$ be the cause-specific hazard function for drug B at time *t*. The null hypothesis under consideration for the log-rank test is *H*_0_:

$$ {H}_0:{\lambda}_1^A(t)={\lambda}_2^B(t) $$

Let ${F}_1^A(t)$ and ${F}_2^B(t)$ be the CIF of recrudescence for drug A and drug B respectively at time *t*. The null hypothesis under consideration for the Gray’s *k*-sample test is *I*_0_:

$$ {I}_0:{F}_1^A(t)={F}_2^B(t) $$

The following hazard ratio $\left({\theta}_{rc}=\frac{\lambda_1^A(t)}{\lambda_2^B(t)}\right)$ of recrudescence (RC) for drug A relative to drug B was assumed:

- *θ*_*rc*_= 1.00 drug B has the same effect on RC as drug A
- *θ*_*rc*_ = 2.72 drug B is associated with increased hazard of RC compared to drug A
- *θ*_*rc*_ = 0.37 drug B is associated with decreased hazard of RC compared to drug A

Similarly, the following hazard ratio (*θ*_*ni*_) of new infection (NI) for drug A relative to drug B was assumed:

- *θ*_*ni*_= 1.00 drug B has the same effect on NI as drug A
- *θ*_*ni*_= 2.72 drug B is associated with increased hazard of NI compared to drug A
- *θ*_*ni*_= 0.37 drug B is associated with decreased hazard of NI compared to drug A

*θ*_*ni*_= 1.00 represents a null scenario, *θ*_*ni*_ = 2.72 represents a scenario where the new drug has a shorter terminal elimination half-life compared to the existing drug and thus exerts a shorter prophylactic effect, while *θ*_*ni*_ = 0.37 represents a scenario where the new drug is associated with a longer post-treatment prophylaxis than the reference drug.

**Fig. 2:**
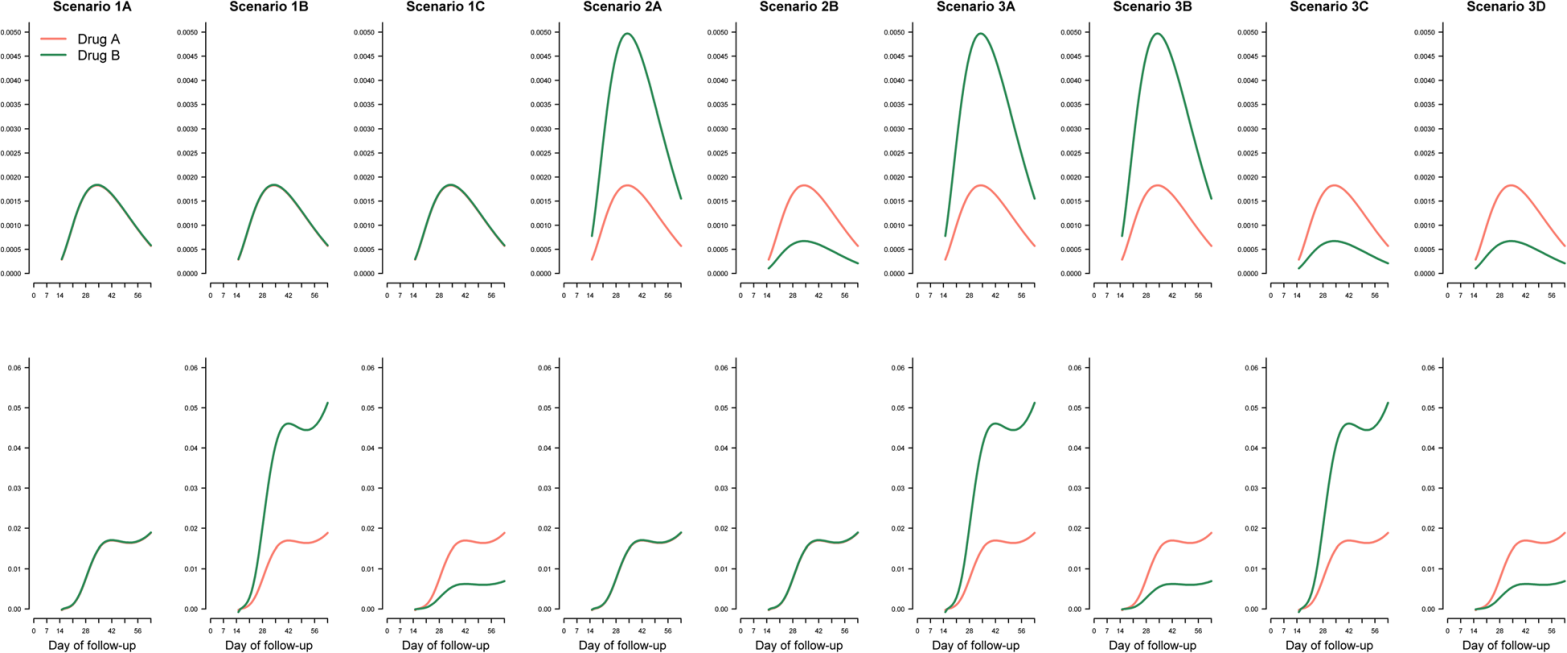
The baseline hazard function for recrudescence and new infection used for simulation study II. Top panel (recrudescence); bottom (new infection). Drug A (orange) is the reference arm and its hazard function for recrudescence and new infection is kept constant across all the simulation scenarios studied. Drug B (green) is a new regimen which is being compared against drug A. Scenario 1 (1A, 1B and 1C) is the null scenario where there is no difference in hazard function of recrudescence between these two drugs. In scenario 2, the two regimens have same hazard function for new infection, but drug B has either increased or decreased hazard of recrudescence with respect to drug A. In scenario 3, the two drugs differ in terms of both recrudescence and new infection

Nine different possible scenarios of drugs A and B were explored in this study (Table 1, Fig. 2). Some of these scenarios presented might not be plausible in antimalarial studies and were kept for completeness as such scenarios might be applicable for other therapeutic interventions [30]. For antimalarial studies, we consider the scenarios where when drug B, compared to drug A exerts unidirectional effect i.e. associated with increased (or decreased) risk of both recrudescence and new infection as the most likely scenario. Similarly, a partially null scenario can be considered likely to be observed in antimalarial trials. For example, when drug A with a short half-life and drug B with a long half-life are compared, then despite observing similar efficacy, it can be expected that more new infections will be observed with drug A (Scenario 1B in Table 1).

**Table 1:** Different scenarios for comparing two drug regimens (drug B compared against drug A) in simulation study II

Scenario	Description
1	**Drug B has same effect on RC as Drug A, and**
1A	Drug B has same effect on NI
1B	Drug B Increases NI
1C	Drug B Decreases NI
2	**Drug B has same effect on NI as Drug A, and**
2A	Drug B increases RC
2B	Drug B decreases RC
3	**Drug B has different effect on both RC and NI relative to Drug A, and**
3A	Drug B increases RC and increases NI
3B	Drug B increases RC and decreases NI
3C	Drug B decreases RC and increases NI
3D	Drug B decreases RC and decreases NI

*RC* Recrudescence, *NI* New infection

Since this simulation was set-up to evaluate type I error when comparing the two drugs, the number of patients needed per arm to detect a difference of a given log-hazard ratio was calculated. A sample size of 500 patients per arm was found to be adequate across all the simulation scenarios studied assuming 80% power for three different log-hazard ratios (Additional file 1, Section 2). However, as for simulation study I, we repeated the simulation for *n* = 100, 200, 500 and 1000 subjects/arm for completeness.

The following steps describe the simulation protocol for each scenario:

i. For each drug arm, time to recrudescence (*t*_1_) was simulated for 500 hypothetical patients using eq. (1). Since drug A is the reference treatment, its intercept parameter was held constant at − 3.7092 for all the simulation scenarios. The intercept parameter for drug B was varied to simulate the scenario of null effect (− 3.7092), increased effect (− 2.7092) or decreased effect (− 4.7092) of drug B on recrudescence relative to drug A. The corresponding hazard functions for different scenarios studied are presented in Fig. 2.
ii. For each drug arm, time to new infection (*t*_2_) was simulated for 500 patients using eq. (2). Since drug A is the reference treatment, its intercept parameter was held constant at − 2.8924 for all the simulation scenarios. The intercept parameter for drug B was varied to simulate the scenario of null effect (− 2.8924), increased effect (− 1.8924) or decreased effect (− 3.8924) of drug B on new infection relative to drug A. The corresponding hazard functions for different scenarios studied are presented in Fig. 2.
iii. Repeat steps (iii-v) as outlined in simulation study I
iv. The difference between drugs A and B in terms of cumulative recrudescence were tested using the log-rank test at day 63 by censoring the new infections. The equality of CIFs for the two regimens was tested using Gray’s *k*-sample test where a new infection was considered a competing risk event. *P*-values and the associated chi-squared test statistic were extracted. The hazard ratio for drug A relative to drug B was estimated using the Cox regression model.
v. The above simulations were repeated 1000 times and the proportion of times the derived *p*-value from log-rank test and Gray’s *k*-sample test was less than 0.05 was calculated. This is equal to the rejection of the null hypothesis that there is no difference between the two treatment regimens in terms of the risk of recrudescence.

### Software

The time to recrudescence and new infection were generated using the **survsim** package in Stata [31] (See Additional file 1, Section 3 for Stata codes). The log-rank test was carried out using the **survdiff** function in the survival package and Gray’s *k*-sample test was performed using the **cuminc** function in the **cmprsk** package in R software (Version 3.2.4) [32].

## Results

### Simulation study I

**Fig. 3:**
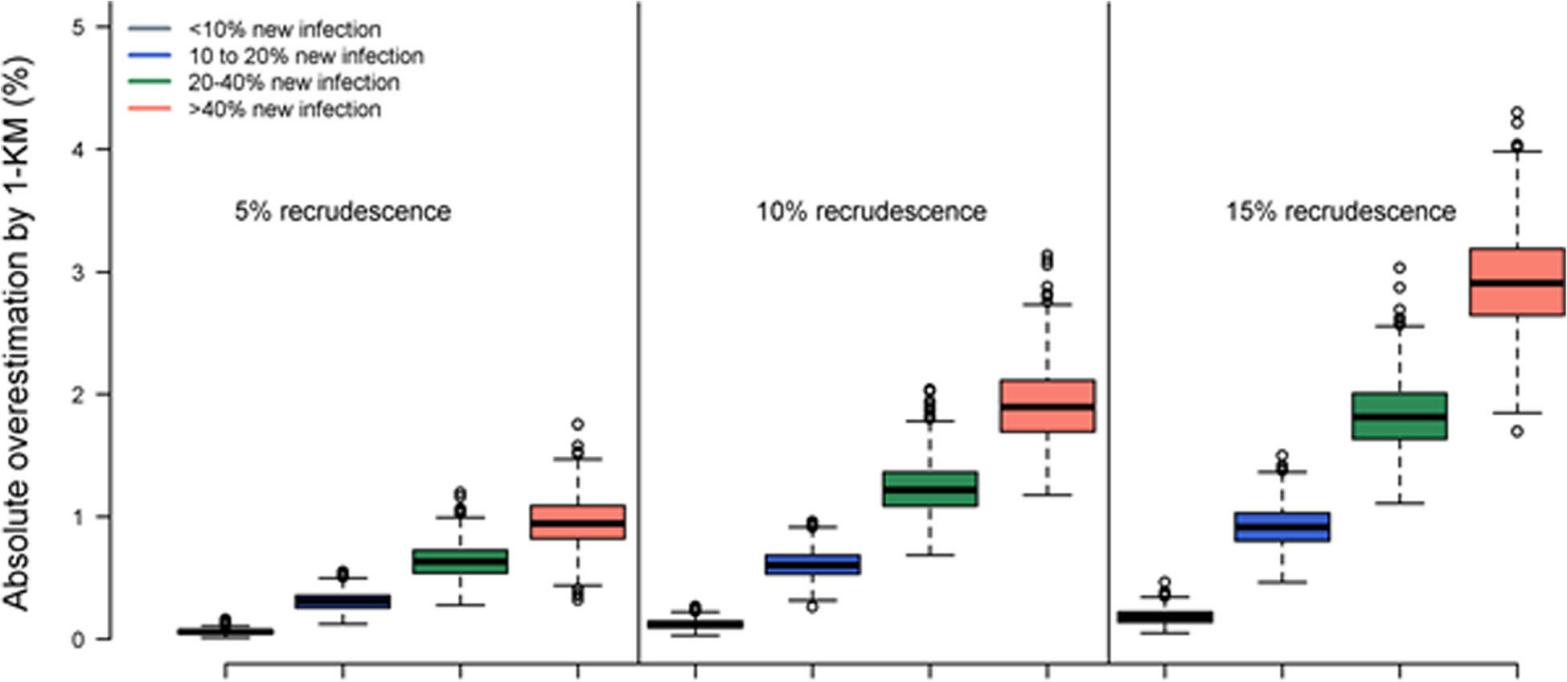
Overestimation of failure using K-M method compared to the CIF in simulation study I (*n* = 500 subjects). The overestimation $\left({\widehat{F}}_{KM}(t)-{\widehat{F}}_{CIF}(t)\right)$ of cumulative recrudescence by the K-M method. Each panel represents different underlying status of drug efficacy on average (~ 5, 10 and 15% recrudescence observed) in a study with a sample size of 500 subjects/trial. The results are presented from 1000 independent simulation runs. The variation in absolute overestimation within each boxplot is due to varying proportion of new infection observed within the simulation scenario. Within each panel, the colours indicate different simulated scenarios of proportions of new infections: < 10% new infections (grey), 10–20% new infections (blue), 20–40% new infections (green) and > 40% new infections (orange), representing areas of progressively increasing malaria transmission from very low to very high

**Fig. 4:**
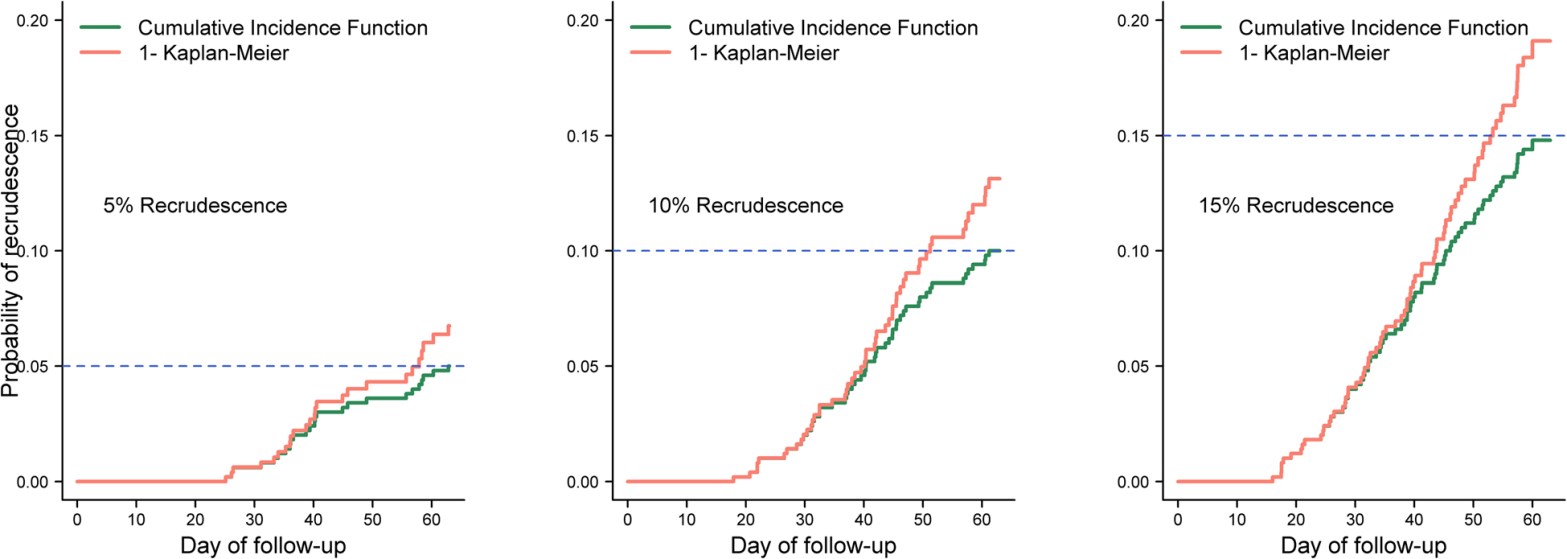
Cumulative failure estimates by study follow-up using extreme examples from simulation study I (*n* = 500 subjects). The figure shows the derived cumulative estimate of recrudescence in three cases from simulation study I where the maximum difference was observed between 1-(K-M) and CIF for 5, 10 and 15% respectively in the areas of very high transmission (> 40% new infections). The absolute difference between the two estimators was 1.8, 3.1 and 4.3% on day 63 respectively for 5, 10 and 15% recrudescence. These three cases are the extreme cases presented in Fig. 3 for the scenarios where > 40% new infections were observed

The findings of this simulation study are presented in Figs. 3 and 4, and Table 2. The 1 minus K-M was associated with an overestimation of cumulative failure in all the scenarios studied. The magnitude of the overestimation increased with i) increasing proportion of new infections, ii) increasing proportion of recrudescences, and iii) the study follow-up duration (Fig. 3).

**Table 2:** Absolute overestimation in cumulative recrudescence by Kaplan-Meier (K-M) method compared to Cumulative Incidence Function (CIF) in simulation study I (*n* = 500 subjects)

		Median absolute overestimation [IQR; Range]		
5% recrudescence	Observed proportion of new infections^a^	Day 28	Day 42	Day 63
< 10% NI	3.8% [1.0–6.6]	0.00% [0.00–0.00; Range:0.00–0.01]	0.02% [0.01–0.02; Range:0.00–0.06]	0.06% [0.05–0.07; Range:0.01–0.16]
10–20% NI	17.0% [12.8–19.8]	0.00% [0.00–0.01; Range:0.00–0.02]	0.08% [0.07–0.10; Range:0.01–0.22]	0.31% [0.26–0.36; Range:0.13–0.55]
20–40% NI	31.2% [25.0–37.8]	0.01% [0.00–0.01; Range:0.00–0.04]	0.18% [0.14–0.22; Range:0.04–0.42]	0.63% [0.54–0.73; Range:0.28–1.20]
40 + % NI	43.0% [40.0–50.0]	0.01% [0.01–0.02; Range:0.00–0.06]	0.28% [0.23–0.34; Range:0.09–0.60]	0.94% [0.82–1.09; Range:0.32–1.75]
10% recrudescence				
< 10% NI	3.6% [1.2–6.2]	0.00% [0.00–0.00; Range:0.00–0.02]	0.03% [0.02–0.04; Range:0.00–0.11]	0.12% [0.10–0.15; Range:0.03–0.27]
10–20% NI	16.4% [10.8–19.8]	0.01% [0.00–0.01; Range:0.00–0.05]	0.17% [0.14–0.21; Range:0.05–0.36]	0.60% [0.53–0.68; Range:0.26–0.96]
20–40% NI	30.0% [24.4–36.2]	0.02% [0.01–0.02; Range:0.00–0.07]	0.36% [0.31–0.42; Range:0.13–0.89]	1.22% [1.09–1.37; Range:0.69–2.04]
40 + % NI	42.0% [40.0–48.0]	0.03% [0.02–0.04; Range:0.00–0.08]	0.56% [0.48–0.65; Range:0.28–1.07]	1.90% [1.69–2.11; Range:1.18–3.13]
15% recrudescence				
< 10% NI	3.4% [1.0–6.2]	0.00% [0.00–0.00; Range:0.00–0.02]	0.05% [0.03–0.07; Range:0.00–0.16]	0.18% [0.14–0.22; Range:0.05–0.46]
10–20% NI	16.0% [10.0–19.8]	0.01% [0.01–0.02; Range:0.00–0.06]	0.26% [0.22–0.31; Range:0.10–0.54]	0.92% [0.80–1.03; Range:0.46–1.50]
20–40% NI	28.8% [23.0–36.6]	0.02% [0.02–0.03; Range:0.00–0.08]	0.54% [0.46–0.62; Range:0.25–1.02]	1.81% [1.64–2.01; Range:1.11–3.03]
40 + % NI	41.0% [40.0–45.8]	0.04% [0.03–0.06; Range:0.00–0.14]	0.88% [0.77–1.00; Range:0.44–1.60]	2.91% [2.64–3.18; Range:1.69–4.30]

^a^Values presented are median [Range]; NI = New infections

In the areas of low transmission (< 10% observed new infection), the maximum overestimation in the derived cumulative risk of recrudescence on day 63 was 0.16% when drug exhibited 95% efficacy (base case scenario), however as the drug efficacy fell to 85%, the difference in estimates increased to 0.46%. In the high transmission areas (> 40% new infections), the maximum absolute overestimation by the 1-KM method was 1.75% for the base case simulation and this rose to 3.13 and 4.30% when the drug efficacy declined to 90 and 85% respectively (Table 2, Fig. 4).

The results when expressed on relative scale exhibited the same trend and conclusion as observed on the absolute scale (Additional file 1, Section 4). The results remained unaffected when the simulation was repeated with sample sizes of *n* = 100, 200, and 1000 patients (Additional file 1, Section 4).

### Simulation study II

**Fig. 5:**
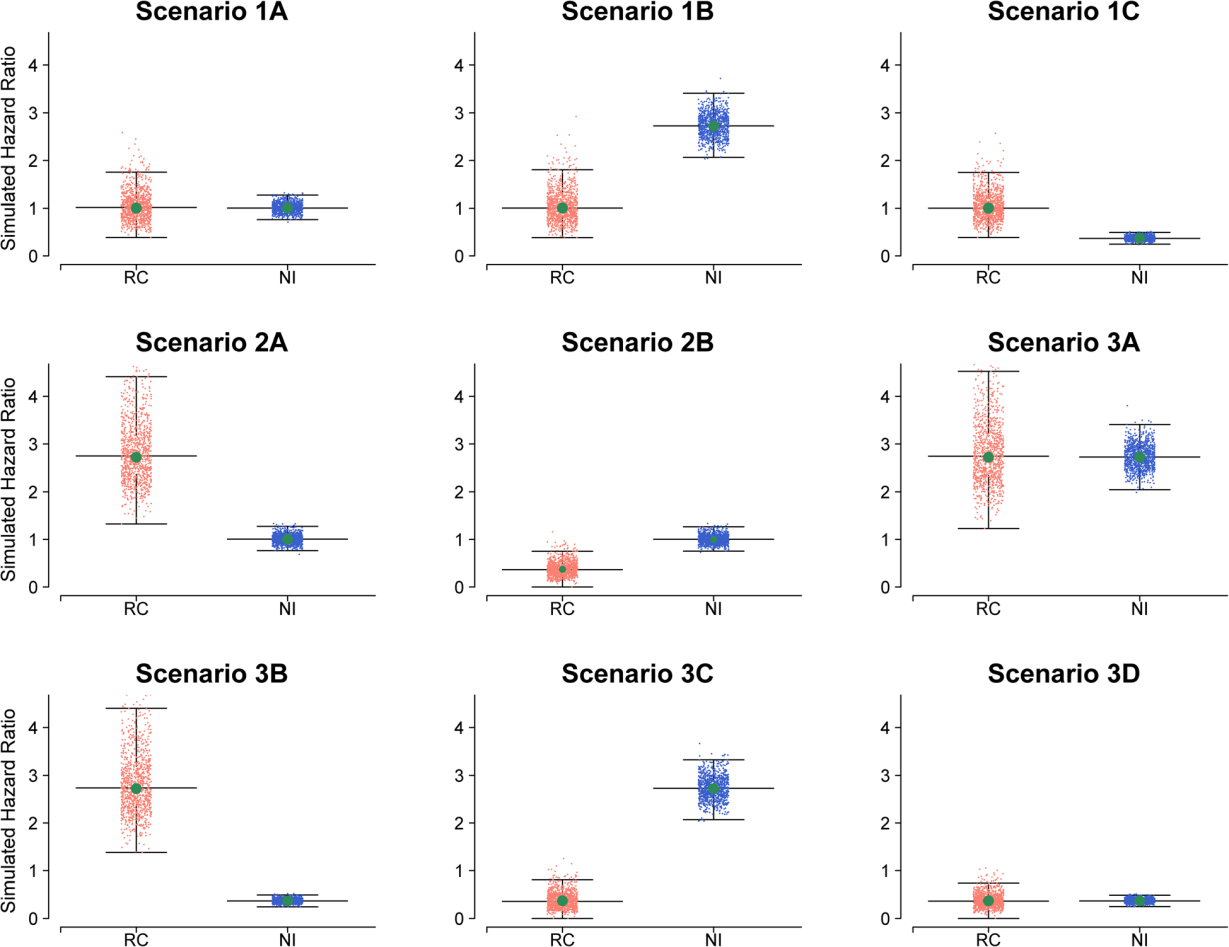
Distribution of simulated hazard ratio (n = 500 subjects) in simulation study II. The scatterplot of estimated hazards ratio for recrudescence and new infection for drug B relative to drug A from 1000 simulation runs. The median and interquartile range is shown. The centre green dot depicts the true hazard ratio which was used to simulate the respective datasets (1, 2.72 or 0.37). RC = recrudescence, NI = New infection. The description of each of the individual scenario is provided in Table 1

For each simulated dataset, the hazard ratio of recrudescence and new infection (for drug B relative to drug A) was estimated using the Cox model with treatment group as a covariate. The distribution of hazard ratios from 1000 simulations is presented in Fig. 5. Table 3 presents the results for the different scenarios considered with sample size of 500 patients per arm, which had at least 80% power to detect the desired hazard ratio for recrudescence between the two drugs across all the scenarios studied.

**Table 3:** Probability of rejecting the null hypothesis at two sided 0.05 level (*n* = 500 subjects per arm) in simulation study II

Scenario	True effect size from which data was simulated ^a^	Median observed proportions of RC and NI in drug A ^b^	Median observed proportions of RC and NI in drug B ^b^	Rejection probability from 1000 simulation runs (10,000 simulation runs)	
1. Drug B has same effect on RC as Drug A				Log-rank test	Gray’s *k*-sample test
A. Drug B has same effect on NI	*HR*_*rc*_ = 1.00, *HR*_*ni*_ = 1.00	2.5% RC; 21.4% NI	2.5% RC; 21.4% NI	0.047 (0.045)	0.0470 (0.045)
B. Drug B Increases NI	*HR*_*rc*_ = 1.00, *HR*_*ni*_ = 2.72	2.5% RC; 21.4% NI	1.9% RC; 38.6% NI	0.052 (0.048)	0.119 (0.125)
C. Drug B Decreases NI	*HR*_*rc*_ = 1.00, *HR*_*ni*_ = 0.37	2.5% RC; 21.4% NI	2.8% RC; 9.4% NI	0.045 (0.047)	0.062 (0.062)
2.Drug B has same effect on NI as Drug A					
A. Drug B increases RC	*HR*_*rc*_ = 2.72, *HR*_*ni*_ = 1.00	2.5% RC; 21.4% NI	6.5% RC; 20.0% NI	0.991 (0.996)	0.995 (0.996)
B. Drug B decreases RC	*HR*_*rc*_ = 0.37, *HR*_*ni*_ = 1.00	2.5% RC; 21.4% NI	0.9% RC; 22.0% NI	0.801 (0.797)	0.804 (0.797)
3. Drug B has different effect on both RC and NI relative to Drug A					
A. Drug B increases RC and increases NI	*HR*_*rc*_ = 2.72, *HR*_*ni*_ = 2.72	2.5% RC; 21.4% NI	5.1% RC; 36.3% NI	0.991 (0.990)	0.897 (0.896)
B. Drug B increases RC and decreases NI	*HR*_*rc*_ = 2.72, *HR*_*ni*_ = 0.37	2.5% RC; 21.4% NI	7.2% RC; 8.7% NI	0.996 (0.723)	0.999 (1.000)
C. Drug B decreases RC and increases NI	*HR*_*rc*_ = 0.37, *HR*_*ni*_ = 2.72	2.5% RC; 21.4% NI	0.7% RC; 39.5% NI	0.714 (0.723)	0.903 (0.910)
D. Drug B decreases RC and decreases NI	*HR*_*rc*_ = 0.37, *HR*_*ni*_ = 0.37	2.5% RC; 21.4% NI	1.0% RC; 9.6% NI	0.828 (0.820)	0.713 (0.718)

^a^Hazard ratio for recrudescence and new infections derived as the ratio of the respective cause-specific hazard function (Fig. 5.6)

*HR*_rc_ Hazard ratio for recrudescence for drug B relative to drug A

*HR*_ni_ Hazard ratio for new infection for drug B relative to drug A

^b^median observed proportion from 1000 simulation runs

*RC* Recrudescence, *NI* New infection

#### No difference in recrudescence

In the null situation (Scenario 1A), where it was postulated there was no difference in the risk of recrudescence and risk of new infection between the two drug regimens, both tests achieved their correct size (*α*) i.e. rejection rate was close to nominal 5%, as expected. Despite there being no difference between the two drugs for both events (as the respective hazard functions for recrudescence and new infections were identical for both drugs), stochastic variations will lead to a rejection of the null hypothesis approximately 5% of the time when the converse is true. In the partially null scenario of 1C i.e. drug B had the same effect on recrudescence as drug A but was associated with decreased hazard of new infection, both tests achieved their correct *α*. In partially null Scenario 1B, where drug B was associated with increased risk of new infection by a hazard ratio of 2.72, the log-rank test correctly achieved its nominal size (5% rejection), but the Gray’s *k*-sample test led to a slightly higher rejection rate (11.9%).

#### Drug A and B have the same post-treatment prophylaxis

When there was no difference between the drug A and drug B in terms of their post-treatment prophylaxis, but drug B was associated with increased recrudescence with a hazard ratio of 2.72 (Scenario 2A), both tests had similar rejection probability. The median proportion of recrudescence observed in this scenario was 6.5% in drug B compared to 2.5% for drug A. In scenario 2B, where the drug B decreased recrudescence relative to drug A (hazard ratio = 0.37), both tests led to rejection of the null hypothesis 80% of the time.

**Fig. 6:**
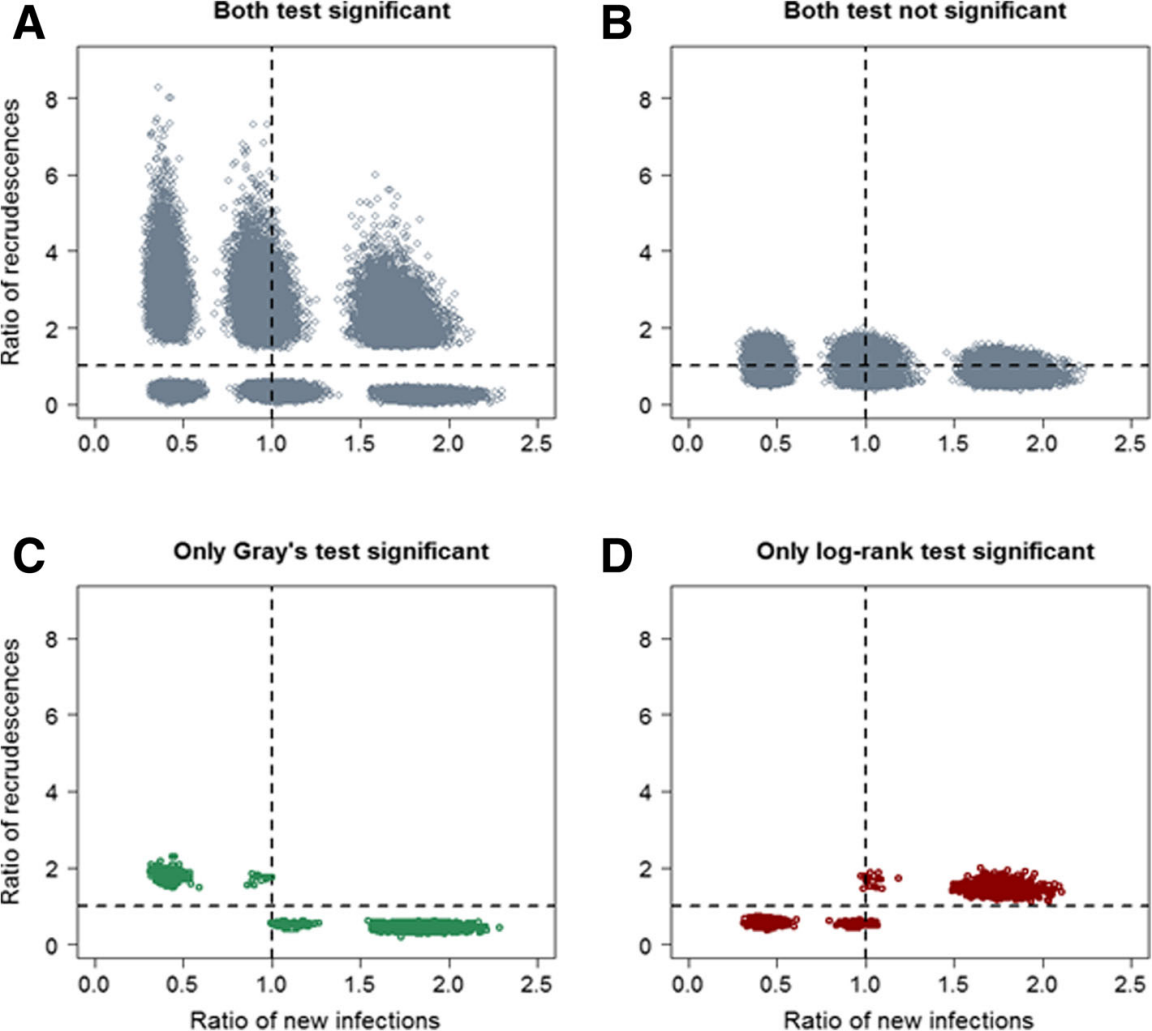
Ratio of recrudescence and new infection in simulation study II (n = 500 subjects/arm). The ratio of recrudescence for drug B relative to drug A plotted against the ratio of new infection for drug B relative to drug A for 1000 simulated dataset

The most relevant and biologically plausible scenario in an antimalarial trial occurs when a new treatment exerts unidirectional effect on recrudescence and new infection (compared to the reference drug), corresponding to scenarios 3A and 3D. In scenario 3A, where drug B was associated with approximately 2-fold increase in both recrudescence and new infection compared to drug A, the log-rank test appeared to be the more powerful of the two approaches with rejection probability of 99% compared to 90% with Gray’s *k*-sample test. In situation 3D, where drug B was associated with a median reduction in recrudescence and new infection by approximately 60%, the log-rank test again proved to be superior by rejecting the null hypothesis of no difference (between drug A and drug B) 82.8% of the time compared to 71.3% by the Gray’s *k*-sample test (Fig. 6, Panel D). The most interesting difference was observed when drug B exerted a differential effect on recrudescence and new infection, i.e. reduced recrudescence but increased new infection compared to drug A (Scenario 3C). In this situation, the Gray’s *k*-sample test appeared to be the more powerful of the two tests (Fig. 6, Panel C). In Scenario 3B, where drug B was associated with increased recrudescence but reduced new infection, the results of the two tests were again very similar.

#### Assumption of proportional hazards

In the simulation scenarios studied, the assumption of proportional hazards was violated in 5.4% (490/9000) of the simulated datasets for the comparison of recrudescence, and 4.5% (407/9000) for new infection. The violation of this assumption didn’t seem to affect the results of the tests as the proportion of times this assumption was violated were similar across different scenarios (Additional file 1, Section 5). Increasing the number of simulation runs to 10,000 from 1000 didn’t change the result (Table 3, results from 10,000 simulation runs shown in parenthesis). However, there were small variations in the results when the simulation was repeated with different sample sizes (Table 4).

**Table 4:** Probability of rejecting the null hypothesis at two sided 0.05 level for different sample sizes in simulation study II

	*n* = 100 subjects per arm		*n* = 200 subjects per arm		*n* = 500 subjects per arm		*n* = 1000 subjects per arm	
Scenario	LR	G	LR	G	LR	G	LR	G
1A	0.043	0.042	0.055	0.045	0.047	0.047	0.042	0.040
1B	0.043	0.055	0.052	0.082	0.052	0.119	0.051	0.217
1C	0.041	0.052	0.047	0.052	0.045	0.062	0.044	0.080
2A	0.554	0.548	0.846	0.838	0.997	0.995	1.000	1.000
2B	0.198	0.187	0.391	0.395	0.801	0.804	0.982	0.983
3A	0.501	0.312	0.787	0.543	0.991	0.897	1.000	0.997
3B	0.570	0.653	0.854	0.911	0.996	1.000	1.000	1.000
3C	0.151	0.251	0.328	0.501	0.714	0.903	0.964	0.996
3D	0.231	0.168	0.422	0.353	0.828	0.713	0.988	0.959

*LR* Log-rank test, G Gray’s *k*-sample test

#### Impact of sample size

In studies with *n* = 100, and 200 (which were known to be under-powered from the sample size calculations), both tests achieved their nominal 5% level i.e. rejection probability close to 5% for scenario 1 (Table 4). In scenarios 2 and 3, where the hazards ratio for recrudescence between the two drugs was 2.72 and 0.37, the rejection probability did not reach the required level of 0.8.

As expected, when the sample size was increased to 1000 patients per arm, both tests achieved their nominal size in the null scenario with the exception of Gray’s *k*-sample test for scenario 1B, which rejected the null hypothesis 21.7% despite there being no difference between the two drugs. In this scenario, the influence of sample size was apparent as the rejection probability using Gray’s *k*-sample test progressively increased with an increase in study sample size. Both tests rejected the null hypothesis in nearly all simulations for scenarios 2 and 3.

## Discussion

Competing risk survival analysis is increasingly being used in the medical and statistical literature [8, 33]. However, this approach remains novel in the context of antimalarial research [34]. The K-M method is the currently recommended approach for deriving antimalarial drug efficacy of uncomplicated *P. falciparum* malaria. Theoretically, the K-M method overestimates the cumulative incidence of recrudescence in the presence of new infection [17]. The magnitude of this overestimation is currently not documented and the implications for comparative efficacy studies is unknown. In order to fill this research gap, we carried out two simulation studies using biologically plausible survival functions consistent with the underlying pharmacokinetics profile of the antimalarial drugs.

The first simulation study quantified the degree of overestimation in cumulative incidence of recrudescence using the naïve 1 minus K-M method compared to the CIF in a single-armed antimalarial trial. The magnitude of the overestimation was found to increase with the increasing proportion of recrudescence, new infection and study follow-up duration; a finding consistent with the statistical and medical literature [16, 17]. The simulation study suggested that the estimates from the two approaches differed by less than 0.1% for most of the scenarios presented in Table 2; such differences are unlikely to have clinical consequences. In a scenario which reflected the current observations of drug efficacy with artemisinin combination therapies (> 95%), the overestimation was negligible in the areas of low transmission intensities, i.e. new infections lower than 10% (Table 2). For high transmission areas, this reached a maximum of 1.75%. However, we have also clearly identified several scenarios where the two methods will lead to a substantially different estimate. The magnitude of the overestimation was greatly increased when antimalarial drug efficacy began to decline. At 90% drug efficacy, the absolute deviation in derived estimates reached a maximum of 0.27% in the areas of low transmission and 3.13% for high transmission areas. When the efficacy fell to the low level of 85%, the overestimation reached 4.30% in the areas of high transmission. Similarly, in antimalarial studies, additional treatment is administered on detecting a recurrent parasitaemia. In such a scenario where the recurrence is due to a new infection, which has masked an existing low-density parasitaemia of the original infection (recrudescence), this would prevent the potential recrudescence from being observed due to additional antimalarial drugs. This will lead to an underestimation of failure. Taken together, our results highlight that estimation of drug failure in areas of high transmission requires careful attention and the CIF provides an alternative approach for deriving the failure estimates.

The second simulation study explored the results from the log-rank test for comparing the cause-specific hazard rates and Gray’s *k*-sample test for comparing the cumulative incidences in comparative drug trials. A total of nine different hypothetical scenarios on how a new drug B might affect the recrudescence and new infection compared to an existing drug A were explored (Table 1). There were contrasting differences in two out of the nine scenarios. When drug B, compared to drug A, was associated with increased (or decreased) risk of both recrudescence and new infection, we found that log-rank test was more powerful compared to Gray’s *k*-sample test for detecting differences between the two treatments. However, when drug B had higher risk of recrudescence and lower risk of new infection (or vice versa) compared to drug A, then Gray’s *k*-sample test was more powerful in detecting the differences between the two drugs in terms of primary endpoint (Table 3). This finding is consistent with the results reported by two previous simulation studies in statistical literature [18, 30]. However, it must be stressed that the latter scenario is less likely to be observed within the context of comparing antimalarial regimens in a real-life situation.

Our simulation study has a number of methodological limitations. First, time to recrudescence and new infection were generated assuming independence. While this greatly simplified the simulation settings, this is an assumption unlikely to be verified and carrying out simulation studies accounting for correlation between recrudescence and new infections remained beyond the scope of this work. Second, we assumed no losses to follow-up for simplicity. A loss to follow-up of approximately 20% is anticipated in antimalarial studies and this can be incorporated in the simulation studies as future work. Third, when simulating time to recrudescence, we used rejection sampling and kept the first 1000 observations with 4–6%, 9–11% and 14–16% recrudescence for the scenarios of 5, 10 and 15% recrudescence, respectively. This approach might have led to less variability between the 1000 simulated datasets. Fourth, in simulation study II, we simulated data based on reference drug A assuming low failure in the areas of low transmission (2.5% recrudescence and 21.4% new infections). Hence, the generalisability of results for comparative studies in areas of different transmission settings might be limited. And finally, this manuscript has focused on the point estimation of the derived failure estimates. However, we would like to emphasise that the uncertainty around the point estimates (associated 95% confidence interval) be given as equal importance as the point estimate.

Our results have important clinical consequences. The current WHO strategy for monitoring and evaluation of antimalarial drug efficacy uses a series of threshold-based approaches. For new drugs to be eligible for introduction as a first line treatment, derived failure estimates should be less than 5%, and for current first line treatments, the failure estimates should not exceed 10% [35]. The results presented in Fig. 4 highlighted the implications for drug policy usage when the derived estimates are at the cusp of these thresholds. The derived estimate of cumulative failure was greater than 5% (Fig. 4a) and 10% (Fig. 4b) when the K-M method was used, but remained below 5 and 10% respectively when using the competing risk survival analysis approach, i.e. the CIF. This highlights that ignoring the competing risk of new infections can result in potentially misleading conclusions being drawn from a clinical study, particularly in high transmission settings where a large fraction of patients may develop new infections during the follow-up period, thus confounding the derived efficacy estimates. Similarly, the effect of competing events has implications for not only standalone trials but also comparative drug trials, particularly when the partner component of the artemisinin combination therapies are eliminated at different rates. For example, lumefantrine, the partner drug in artemether-lumefantrine (AL), has an elimination half-life of 4 days and hence almost all antimalarial activity is sub-therapeutic within 16 days [36]. Conversely the elimination half-life of piperaquine (partner drug in dihydroatemsinin-piperaquine (DP)) is four weeks and it exerts prolonged post treatment prophylaxis, reducing the risk of recurrent infections for up to 42 days [36]. Hence, the observed proportion of competing risk events is expected to be significantly lower following DP compared to AL, especially in the areas of high transmission. When a large fraction of patients develop new infections, fewer patients are available from which recrudescences can be observed. Hence, it is important that the proportion of competing risk events be taken into consideration when comparing two regimens with different pharmacological properties.

There is an ongoing debate in medical and statistical literature regarding the choice of the method for comparing treatment regimens in the presence of competing risk events [19, 30, 37–39]. It is increasingly being advocated that if the research interest is in understanding the biological mechanism of how a treatment affects hazard rate, the log-rank test is considered the appropriate method. However, when the interest is in comparison of overall risk i.e. if individuals receiving a particular drug are more likely to experience recrudescence, the comparison of CIF through Gray’s *k*-sample test is considered appropriate [17, 40, 41]. Many authors advocate presenting results of both these approaches to provide a complete biological understanding of the treatment on different endpoints [17, 42]. It is important that researchers are aware that the choice of the analytical method in the presence of competing risk events should be guided by the research question of interest.

## Conclusions

Our simulation study showed that 1 minus K-M method led to an overestimation of cumulative antimalarial treatment failure compared to the CIF and the degree of overestimation was far greater in high transmission areas. In the areas where a large proportion of recurrences are attributable to new infections, the use of CIF should be considered as an alternative approach for the derivation of failure estimates for antimalarial studies. For comparative studies of antimalarial treatments, the choice of the statistical test should be guided by whether the rate or cumulative risk of recrudescence is the outcome of interest.

## Additional file

Additional file 1: Additional text and results (DOCX 130 kb)

## Abbreviations

${\widehat{S}}_{KM}(t)$: Kaplan-Meier estimates of drug efficacy at time *t*
${\widehat{F}}_{KM}(t)$: The complement of Kaplan-Meier estimate$\left[1-{\widehat{S}}_{KM}(t)\right]$
CBH: Cumulative baseline hazard
CIF: Cumulative Incidence Function
HR: Hazards Ratio
K-M: Kaplan-Meier
NI: New infection
RC: Recrudescence
WHO: World Health Organization
