# Supplementary material for: Evaluating antimalarial efficacy in single-armed and comparative drug trials using competing risk survival analysis: a simulation study
Source: BMC Med Res Methodol. 2019 May 17;19:107. doi: 10.1186/s12874-019-0748-2 (PMC6525412; doi:10.1186/s12874-019-0748-2)
Supplement: Supplementary file 1 — Additional text and results (DOCX 130 kb) [file 12874_2019_748_MOESM1_ESM.docx]

**Additional file to: Evaluating antimalarial efficacy in single-armed and comparative drug trials using competing risk survival analysis: A simulation study**

Prabin Dahal ^1,2^*, Philippe J. Guerin^1,2^, Ric N. Price^1,2,3^, Julie A. Simpson^4^, Kasia Stepniewska^1,2^

^1^WorldWide Antimalarial Resistance Network (WWARN), Oxford, UK

^2^Centre for Tropical Medicine and Global Health, Nuffield Department of Clinical Medicine, University of Oxford, Oxford, UK

^3^Global and Tropical Health Division, Menzies School of Health Research and Charles Darwin University, Darwin, Australia

^4^Centre for Epidemiology and Biostatistics, Melbourne School of Population and Global Health, The University of Melbourne, Melbourne, Australia

1. **Cause-specific and sub-distribution hazard**

## Cause-specific hazard

The cause-specific hazard defines the instantaneous risk of an event occurring per unit time for the specific cause of the event, among subjects without any prior events. This is the probability of failure due to cause *k* (for example, recrudescence) at time *t*, given that no failures of any kind (neither recrudescence nor new infection) have occurred thus far. The cause-specific hazard at a given time point for an event type can be computed by dividing the number of individuals experiencing that event type by the total number of individuals who were in the risk-set immediately prior to the time *t*. Individuals are removed from the risk-set if they experience either of the two events or those who are lost to follow-up.

## The sub-distribution hazard

The sub-distribution hazard function, introduced by Fine and Gray [1], is the probability of observing an event *k* in the next time interval, given that no cause *k* failures (neither recrudescence nor new infection) have occurred thus far. The sub-distribution hazard at a given time-point for an event type can be computed by dividing the number of individuals experiencing the event type (e.g. recrudescence) by the total number individuals who were in the risk-set immediately prior to the time *t*, while still maintaining individuals who experienced competing events in the risk-set. This is the main difference between cause-specific hazard and the sub-distribution hazard approach (Figure 5.1). Maintaining subjects with a new infection in the risk-set allows them to act as a place holder that represents the proportion of the risk-set that can never experience the primary outcome [2]. Like with the cause-specific hazard function, those who are lost to follow-up are removed from the risk-set.

1. **Sample size calculation for Simulation Study II**

The simulation study was set-up to compare two drugs. Based on the hazard ratio (of 2.72 and 0.37) used to simulate the data, the number of patients needed per arm to detect a difference of a given log-hazard ratio ($\theta_{R})$ was calculated in a two-step procedure. The sample size calculation was performed following Collett (2015) [3]. The equation outlined in Step I below is presented as equation 15.1 in page 473 of Collett (2015) [3], and for Step II the presented equation corresponds to equation 15.11 in page 479 of Collett (2015) [3].

**Step I:** In the first step, the number of events required per arm to detect the desired log-relative risk ($\theta_{R})$ was calculated. The number of recrudescences (d) required per group assuming a one-to-one treatment allocation ratio was calculated using:

$$d=\frac{4{(Z_{\alpha/2}+Z_{\beta})}^{2}}{{\theta_{R}}^{2}}$$

where $Z_{\alpha/2}$ and $Z_{\beta}$are the upper $\alpha/2$- and $\beta$-points of the standard normal distribution, and $\theta_{R}$ is the log-hazard ratio.

**Step II:** In the second step, the total number of patients (N) required per arm is computed by dividing the number of events computed in Step I by the overall probability of observing recrudescence during the study follow-up. For accrual duration *a*, and follow-up period *f*, the overall probability of observing recrudescence was computed as:

$$P\left( d \right)=\frac{1}{6}\{\bar{S}\left( f \right)+4\bar{S}\left( 0.5a+f \right)+\bar{S}\left( a+f \right)\}$$

where $\bar{S}\left( f \right)$ is the average of the estimated value of survival functions of two drugs. The accrual duration for antimalarial studies are much longer than the follow-up period with studies often running for two to three malaria seasons. The follow-up (*f*) period, in contrast lasts only up to 9 weeks. For simplicity, *a* was assumed to be 0. The sample size required per group to detect a statistically significant difference for a given log-hazards ratio was then obtained as:

$$N=\frac{\left( \frac{4\left( Z_{\frac{\alpha}{2}}+Z_{\beta} \right)^{2}}{{\theta_{R}}^{2}} \right)}{1-\bar{S}\left( f \right)}$$

In simulation study II, the assumed efficacy for drug A (reference), $S_{A}(t)$ on day 63 was 0.940. The assumed efficacies for drug B, $S_{B}(t)$, for different scenarios together with the sample size required to detect the given hazards ratio with 80 and 90% power are presented below:

|  |  |  |  |  | **80% power** | | **90% power** | |
| --- | --- | --- | --- | --- | --- | --- | --- | --- |
| Scenario | $S_{A}(t)$ | $S_{B}(t)$ | $1-\bar{S}\left( t \right)$ | HR to detect | Events/arm  required | SS/arm  required | Events/arm  required | SS/arm  required |
| 1A | 0.94 | 0.94 | 0.06 | 1 | - | - | - | - |
| 1B | 0.94 | 0.94 | 0.06 | 1 | - | - | - | - |
| 1C | 0.94 | 0.94 | 0.06 | 1 | - | - | - | - |
| 2A | 0.94 | 0.84 | 0.11 | 2.72 | 16 | 146 | 21 | 195 |
| 2B | 0.94 | 0.98 | 0.04 | 0.37 | 16 | 384 | 21 | 514 |
| 3A | 0.94 | 0.84 | 0.11 | 2.72 | 16 | 146 | 21 | 195 |
| 3B | 0.94 | 0.84 | 0.11 | 2.72 | 16 | 146 | 21 | 195 |
| 3C | 0.94 | 0.84 | 0.11 | 0.37 | 16 | 147 | 21 | 197 |
| 3D | 0.94 | 0.98 | 0.04 | 0.37 | 16 | 384 | 21 | 514 |

$S_{A}(t)$= Survival estimate at the end of study follow-up for drug A

$S_{B}(t)$= Survival estimate at the end of study follow-up for drug B

$\bar{S}\left( t \right)$ = average survival between two groups

SS = Sample size

A sample size of 500 patients per am was found to be adequate across all the simulation scenarios studied assuming 80% power.

1. **Stata do-file for generation of the dataset**

**3A: Simulation study I**

*-------------------------------------------------------------------------------------------------------------------

* Title : Competing risk analysis in standalone antimalarial studies

*--------------------------------------------------------------------------------------

* stime : Time to primary event of interest (recrudescence) in days

* stime2 : Time to competing risk event (new infection)in days

* event : Primary event of interest (recrudescence) (1= recrudescence, 0=otherwise)

* event2 : Competing risk event (new infection) (1= new infection, 0=otherwise)

* mintime : Minimum time to recrudescence/new infection. This is set to 14 days.

* maxtime : Minimum time to recrudescence/new infection. This is length of study follow-up and set to 63 days.

*-------------------------------------------------------------------------------------------------------------------

* Version: Stata 15.1

*-------------------------------------------------------------------------------------------------------------------

ssc install survsim

* Generate a trial with 500 patients

set obs 500

* Generate time to recrudescence

survsim stime event, logcumhazard( -3.709159 :- (63.62843 :* (log(#t):^(-1):-0.2849155948)) :-0.3799831 :*((log(#t):^(2) ) :- 12.318776)) mintime(14) maxtime(63)

* Generate time to new infection

survsim stime2 event2, logcumhazard(-5.600422 :+ (9501.215 :* (log(#t):^(-2):-.0858274119)) :- 31651.33 :*((log(#t):^(-2) :* log(log(#t))) :-0.1053710362):+ 29340.83:*((log(#t):^(-2) :* log(log(#t)):^(2)) :-0.1293649082):-12690.51:*((log(#t):^(-2) :* log(log(#t)):^(3)) :-0.1588223868)) mintime(14) maxtime(63)

**3B: Stata do-file for generation of the dataset for simulation study II**

*-----------------------------------------------------------------------------------------------------------

* Title : Competing risk analysis in comparative antimalarial studies

*--------------------------------------------------------------------------------------

* stime_drugA_rc : Time to primary event of interest (recrudescence) in days for drug A

* stime_drugA_ri : Time to competing risk event (new infection) in days for drug A

* event1_A : Primary event of interest (recrudescence) (1= recrudescence, 0=otherwise) for drug A

* event2_A : Competing risk event (new infection) (1= new infection, 0=otherwise) for drug A

*-------------------------------------------------------------------------------------------------------------

* stime_drugB_rc : Time to primary event of interest (recrudescence) in days for drug B

* stime_drugB_ri : Time to competing risk event (new infection) in days for drug B

* event1_B : Primary event of interest (recrudescence) (1= recrudescence, 0=otherwise) for drug B

* event2_B : Competing risk event (new infection) (1= new infection, 0=otherwise) for drug B

*-----------------------------------------------------------------------------------------------------------

* mintime : Minimum time to recrudescence/new infection. This is set to 14 days.

* maxtime : Minimum time to recrudescence/new infection. This is length of study follow-up=63 days.

*----------------------------------------------------------------------------------------------------------------

* Version: Stata 15.1

*----------------------------------------------------------------------------------------------------------------

ssc install survsim

// Generate a trial with 500 patients per treatment arm

set obs 500

// time to recrudescence for reference drug (drug A)

survsim stime_drugA_rc event1_A , logcumhazard( -3.709159 :- (63.62843 :* (log(#t):^(-1):-0.2849155948)) :-0.3799831 :*((log(#t):^(2) ) :- 12.318776)) mintime(14) maxtime(63)

// time to new infection for reference drug (drug A)

survsim stime_drugA_ri event2_A, logcumhazard( -2.892372:+ (9501.215 :* (log(#t):^(-2):-.0858274119)) :- 31651.33 :*((log(#t):^(-2) :* log(log(#t))) :-0.1053710362):+ 29340.83:*((log(#t):^(-2) :* log(log(#t)):^(2)) :-0.1293649082):-12690.51:*((log(#t):^(-2) :* log(log(#t)):^(3)) :-0.1588223868)) mintime(14) maxtime(63)

// time to recrudescence for new drug (drug B)

survsim stime_drugB_rc event1_B, logcumhazard( -3.709159 :- (63.62843 :* (log(#t):^(-1):-0.2849155948)) :-0.3799831 :*((log(#t):^(2) ) :- 12.318776)) mintime(14) maxtime(63)

// time to new infection for new drug (drug B)

survsim stime_drugB_ri event2_B, logcumhazard( -2.892372:+ (9501.215 :* (log(#t):^(-2):-.0858274119)) :- 31651.33 :*((log(#t):^(-2) :* log(log(#t))) :-0.1053710362):+ 29340.83:*((log(#t):^(-2) :* log(log(#t)):^(2)) :-0.1293649082):-12690.51:*((log(#t):^(-2) :* log(log(#t)):^(3)) :-0.1588223868)) mintime(14) maxtime(63)

1. **Further results for simulation study I**


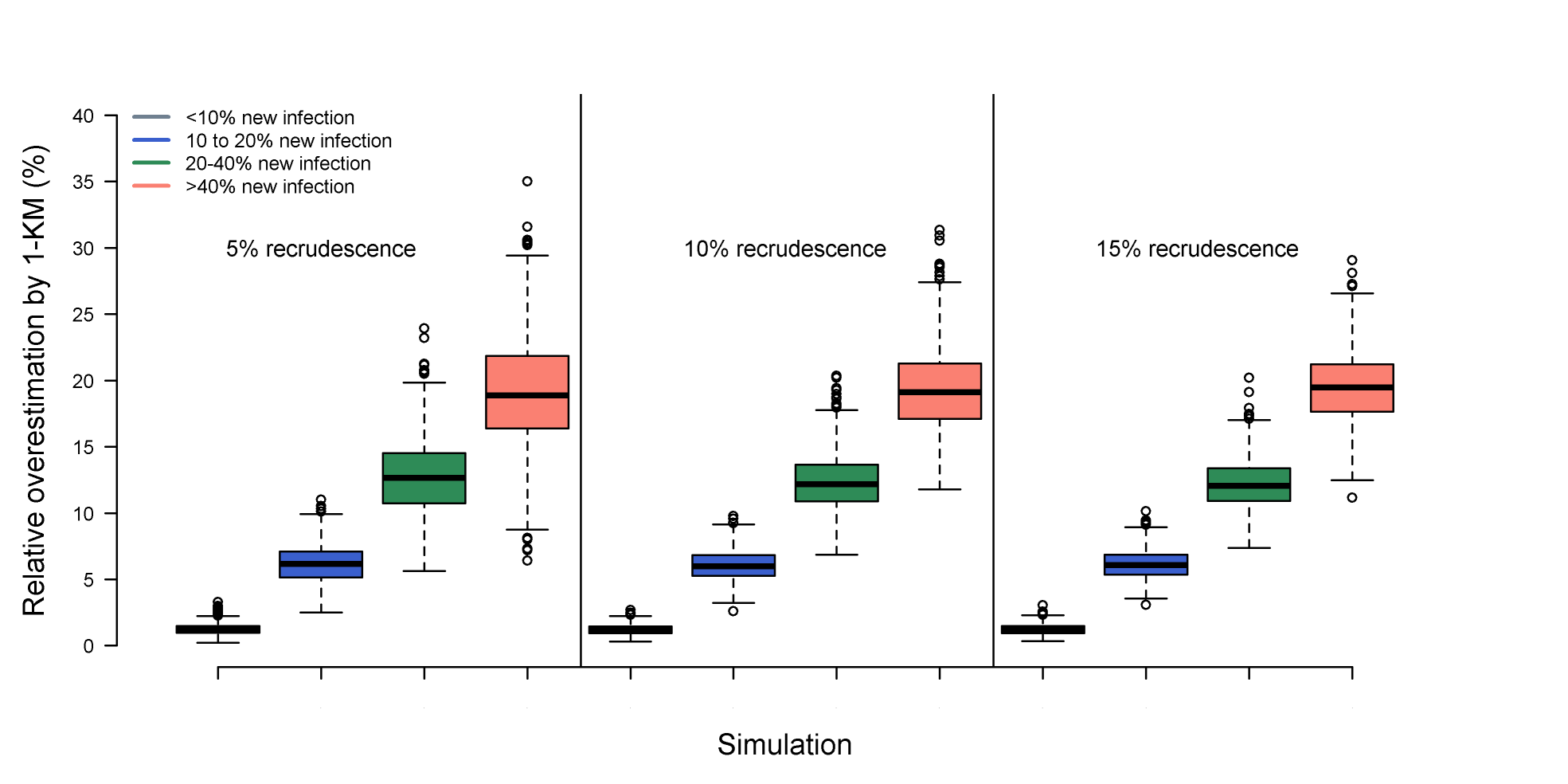


**Figure 1: Relative overestimation of recrudescence failure using Kaplan-Meier (K-M) approach compared to the Cumulative Incidence Function (CIF) in a simulation study assessing standalone efficacy on day 63 (n=500 subjects)**

**Legend**: The overestimation $({\hat{F}_{KM}\left( t \right)-\hat{F}}_{CIF}\left( t \right))$ of cumulative recrudescence using K-M approach. Each panel represents different underlying status of drug efficacy on average (~5%, 10% and 15% recrudescence observed) in a study with a sample size of 500. The results are presented from 1,000 independent simulation runs. The variation in absolute overestimation within each boxplot is due to varying proportion of new infections. Within each panel, the colours indicate different proportion of new infections: <10% new infections (grey), 10-20% new infections (blue), 20-40% new infections (green) and >40% new infections (orange), representing areas of progressively increasing malaria transmission.

Table 1: Absolute overestimation in recrudescence failure by Kaplan-Meier (K-M) method compared to Cumulative Incidence Function (CIF) in simulation study I (n=100 subjects)

|  |  | **Median absolute overestimation[IQR; Range]** | | |
| --- | --- | --- | --- | --- |
| **5% recrudescence** | **Observed proportion of new infections** | **Day28** | **Day 42** | **Day 63** |
| <10% NI | 3% [0 - 9] | 0.00% [0.00 - 0.00 ; Range:0.00-0.04] | 0.01% [0.00 - 0.03 ; Range:0.00-0.15] | 0.05% [0.02 - 0.09 ; Range:0.00-0.34] |
| 10-20% NI | 16% [10 - 19] | 0.00% [0.00 - 0.00 ; Range:0.00-0.08] | 0.08% [0.04 - 0.12 ; Range:0.00-0.39] | 0.30% [0.21 - 0.41 ; Range:0.00-0.79] |
| 20-40% NI | 31% [20 - 39] | 0.00% [0.00 - 0.01 ; Range:0.00-0.12] | 0.18% [0.11 - 0.27 ; Range:0.00-0.89] | 0.66% [0.45 - 0.9 ; Range:0.01-2.05] |
| 40+% NI | 44% [40 - 59] | 0.01% [0.00 - 0.02 ; Range:0.00-0.29] | 0.31% [0.17 - 0.45 ; Range:0.00-1.09] | 1.08% [0.71 - 1.49 ; Range:0.00-3.3] |
| **10% recrudescence** |  |  |  |  |
| <10% NI | 4% [0 - 9] | 0.00% [0.00 - 0.00; Range:0.00-0.05] | 0.02% [0.00 - 0.05 ; Range:0.00-0.29] | 0.11% [0.06 - 0.18 ; Range:0.00-0.53] |
| 10-20% NI | 16% [10 - 19] | 0.00% [0.00 - 0.01; Range:0.00-0.12] | 0.15% [0.09 - 0.22 ; Range:0.00-0.80] | 0.55% [0.41 - 0.72 ; Range:0.00-1.52] |
| 20-40% NI | 30% [20 - 39] | 0.01% [0.00 - 0.02 ; Range:0.00-0.15] | 0.32% [0.20 - 0.47 ; Range:0.00-1.51] | 1.19% [0.90 - 1.53 ; Range:0.12-3.33] |
| 40+% NI | 43% [40 - 56] | 0.02% [0.00 - 0.04 ; Range:0.00-0.23] | 0.58% [0.40 - 0.78 ; Range:0.00-2.58] | 1.97% [1.48 - 2.45 ; Range:0.35-5.58] |
| **15% recrudescence** |  |  |  |  |
| <10% NI | 3% [0 - 9] | 0.00% [0.00 - 0.00 ; Range:0.00-0.09] | 0.03% [0.00 - 0.08 ; Range:0.00-0.40] | 0.16% [0.07 - 0.25 ; Range:0.00-0.66] |
| 10-20% NI | 15% [10 - 19] | 0.00% [0.00 - 0.02 ; Range:0.00-0.15] | 0.22% [0.14 - 0.33 ; Range:0.00-1.04] | 0.82% [0.64 - 1.06 ; Range:0.11-1.93] |
| 20-40% NI | 29% [20 - 39] | 0.02% [0.00 - 0.04 ; Range:0.00-0.25] | 0.52% [0.36 - 0.70 ; Range:0.00-1.65] | 1.73% [1.37 - 2.21 ; Range:0.38-4.56] |
| 40+% NI | 43% [40 - 57] | 0.03% [0.01 - 0.07 ; Range:0.00-0.32] | 0.84% [0.61 - 1.15 ; Range:0.04-2.66] | 2.90% [2.27 - 3.62 ; Range:0.38-6.19] |

NI= New infection; K-M = failure estimate derived using complement of Kaplan-Meier estimate; IQR = Interquartile range; CIF = Cumulative Incidence Function

Table 2: Absolute overestimation in recrudescence failure by Kaplan-Meier (K-M) method compared to Cumulative Incidence Function (CIF) in simulation study I (n=200 subjects)

|  |  | **Median absolute overestimation[IQR; Range]** | | |
| --- | --- | --- | --- | --- |
| **5% recrudescence** | **Observed proportion of new infections** | **Day28** | **Day 42** | **Day 63** |
| <10% NI | 4% [0 - 8] | 0.00% [0.00 - 0.00; Range:0.00-0.02] | 0.01% [0.01 - 0.03 ;Range:0.00-0.09] | 0.06% [0.04 - 0.09 ; Range:0.00-0.23] |
| 10-20% NI | 16.5% [10 - 19.5] | 0.00% [0.00 - 0.01 ; Range:0.00-0.05] | 0.08% [0.05 - 0.10 ; Range:0.00-0.29] | 0.29% [0.22 - 0.36 ; Range:0.00-0.74] |
| 20-40% NI | 31.5% [22 - 39] | 0.01% [0.00 - 0.01 ; Range:0.00-0.09] | 0.18% [0.12 - 0.24 ; Range:0.01-0.83] | 0.62% [0.47 - 0.78 ; Range:0.09-1.37] |
| 40+% NI | 43.5% [40 - 54.5] | 0.01% [0.00 - 0.02 ; Range:0.00-0.09] | 0.28% [0.20 - 0.38 ; Range:0.00-0.8] | 0.98% [0.73 - 1.21 ; Range:0.16-2.79] |
| **10% recrudescence** |  |  |  |  |
| <10% NI | 3.5% [0.5 - 8.5] | 0.00% [0.00 - 0.00 ; Range:0.00-0.04] | 0.03% [0.01 - 0.05 ; Range:0.00-0.23] | 0.11% [0.07 - 0.16 ; Range:0.00-0.40] |
| 10-20% NI | 16% [10.5 - 19.5] | 0.01% [0.00 - 0.01 ; Range:0.00-0.08] | 0.16% [0.12 - 0.22 ; Range:0.00-0.47] | 0.58% [0.47 - 0.69 ; Range:0.09-1.15] |
| 20-40% NI | 30% [21 - 39.5] | 0.01% [0.01 - 0.03 ; Range:0.00-0.09] | 0.36% [0.26 - 0.45 ; Range:0.04-0.92] | 1.24% [1.01 - 1.49 ; Range:0.34-2.8] |
| 40+% NI | 42.5% [40 - 54] | 0.02% [0.01 - 0.04 ; Range:0.00-0.19] | 0.55% [0.44 - 0.70 ; Range:0.12-1.34] | 1.91% [1.57 - 2.22 ; Range:0.63-3.79] |
| **15% recrudescence** |  |  |  |  |
| <10% NI | 3.5% [0 - 8] | 0.00% [0.00 - 0.00 ; Range:0.00-0.05] | 0.04% [0.02 - 0.07 ; Range:0.00-0.25] | 0.17% [0.12 - 0.24 ; Range:0.00-0.63] |
| 10-20% NI | 16% [10 - 19.5] | 0.01% [0.00 - 0.02 ; Range:0.00-0.09] | 0.25% [0.18 - 0.33 ; Range:0.03-0.66] | 0.89% [0.73 - 1.06 ; Range:0.31-1.85] |
| 20-40% NI | 28.5% [20 - 39] | 0.02% [0.01 - 0.04 ; Range:0.00-0.19] | 0.53% [0.41 - 0.65 ; Range:0.11-1.34] | 1.80% [1.51 - 2.11 ; Range:0.70-3.35] |
| 40+% NI | 42% [40 - 49.5] | 0.04% [0.02 - 0.06 ; Range:0.00-0.19] | 0.87% [0.69 - 1.06 ; Range:0.19-2.34] | 2.91% [2.47 - 3.36 ; Range:1.21-5.59] |

NI= New infection; K-M = failure estimate derived using complement of Kaplan-Meier estimate; IQR = Interquartile range; CIF = Cumulative Incidence Function

Table 3: Absolute overestimation in recrudescence failure by Kaplan-Meier (K-M) method compared to Cumulative Incidence Function (CIF) in simulation study I (n=1,000 subjects)

|  |  | **Median absolute overestimation[IQR; Range]** | | |
| --- | --- | --- | --- | --- |
| **5% recrudescence** | **Observed proportion of new infections** | **Day28** | **Day 42** | **Day 63** |
| <10% NI | 3.7% [2 - 5.7] | 0.00% [0.00 - 0.00 ; Range:0.00-0.01] | 0.02% [0.01 - 0.02 ; Range:0.00-0.06] | 0.06% [0.05 - 0.08 ; Range:0.03-0.13] |
| 10-20% NI | 17.2% [13.5 - 19.9] | 0.00% [0.00 - 0.01 ; Range:0.00-0.02] | 0.09% [0.07 - 0.10 ; Range:0.02-0.15] | 0.32% [0.28 - 0.36 ; Range:0.16-0.55] |
| 20-40% NI | 31.2% [26.4 - 36.7] | 0.01% [0.01 - 0.01 ; Range:0.00-0.03] | 0.18% [0.15 - 0.21 ; Range:0.07-0.33] | 0.63% [0.57 - 0.70 ; Range:0.34-0.95] |
| 40+% NI | 42.7% [38.1 - 47.2] | 0.01% [0.01 - 0.02 ; Range:0.00-0.04] | 0.27% [0.24 - 0.32 ; Range:0.11-0.54] | 0.93% [0.83 - 1.04 ; Range:0.57-1.47] |
| **10% recrudescence** |  |  |  |  |
| <10% NI | 3.6% [1.8 - 5.6] | 0.00% [0.00 - 0.00 ; Range:0.00-0.01] | 0.04% [0.03 - 0.04 ; Range:0.01-0.08] | 0.13% [0.11 - 0.15 ; Range:0.05-0.25] |
| 10-20% NI | 16.4% [13.1 - 19.9] | 0.01% [0.01 - 0.01 ; Range:0.00-0.03] | 0.18% [0.16 - 0.20 ; Range:0.08-0.29] | 0.63% [0.57 - 0.69 ; Range:0.34-1.01] |
| 20-40% NI | 30% [25.1 - 34.5] | 0.02% [0.01 - 0.02 ; Range:0.00-0.04] | 0.37% [0.32 - 0.41 ; Range:0.22-0.59] | 1.25% [1.15 - 1.36 ; Range:0.76-1.87] |
| 40+% NI | 41.6% [40 - 46.3] | 0.03% [0.02 - 0.03 ; Range:0.01-0.06] | 0.57% [0.51 - 0.63 ; Range:0.35-0.90] | 1.87% [1.71 - 2.02 ; Range:1.22-2.57] |
| **15% recrudescence** |  |  |  |  |
| <10% NI | 3.5% [1.8 - 6.3] | 0.00% [0.00 - 0.00 ; Range:0.00-0.02] | 0.05% [0.04 - 0.06 ; Range:0.01-0.13] | 0.19% [0.16 - 0.22 ; Range:0.09-0.43] |
| 10-20% NI | 15.8% [12.9 - 19.4] | 0.01% [0.01 - 0.02 ; Range:0.00-0.04] | 0.27% [0.24 - 0.30 ; Range:0.14-0.48] | 0.92% [0.85 – 1.00 ; Range:0.61-1.34] |
| 20-40% NI | 28.6% [24.5 - 33.5] | 0.03% [0.02 - 0.03 ; Range:0.01-0.08] | 0.54% [0.48 - 0.60 ; Range:0.30-0.81] | 1.82% [1.68 - 1.95 ; Range:1.14-2.42] |
| 40+% NI | 40.8% [40 - 44.7] | 0.04% [0.03 - 0.05 ; Range:0.01-0.09] | 0.85% [0.77 - 0.94 ; Range:0.53-1.30] | 2.82% [2.63 - 3.02 ; Range:2.01-3.85] |

NI= New infection; K-M = failure estimate derived using complement of Kaplan-Meier estimate; IQR = Interquartile range; CIF = Cumulative Incidence Function

1. **Further results for simulation study II**

Table 4: The assumption of proportional hazard (PH) in simulated datasets in simulation study II (n=500/arm)

| **Recrudescence** | Assumption of proportional hazard ^a^  Satisfied Violated | | Proportion of times  PH assumption violated |
| --- | --- | --- | --- |
| Both tests significant | 4930 | 280 | 5.4% |
| Only Gray's test significant | 313 | 17 | 5.2% |
| Only log-rank test significant | 252 | 9 | 3.4% |
| Both test not significant | 3015 | 184 | 5.8% |
| Overall | 8510 | 490 | 5.4% |
| **New Infection** |  |  |  |
| Both tests significant | 4961 | 249 | 4.8% |
| Only Gray's test Significant | 315 | 15 | 4.5% |
| Only log-rank test significant | 250 | 11 | 4.2% |
| Both test not significant | 3067 | 132 | 4.1% |
| Overall | 8593 | 407 | 4.5% |

^a^ The assumption of PH tested using cox.zph function in R survival package from a cox model with drug regimen as the only factor. The assumption of PH was defined to be violated if *p*-value <0.05 for the test using cox.zph

1. **References**

1. Fine JP, Gray RJ. A Proportional Hazards Model for the Subdistribution of a Competing Risk. J Am Stat Assoc. 1999;94:196–509.

2. Lau B, Cole SR, Gange SJ. Competing risk regression models for epidemiologic data. Am J Epidemiol. 2009;170:244–56.

3. Collett D. Sample size requirements for a survival study. Model Surviv data Med Res. Third. Boca Raton: Chapman & Hall/CRC Biostatistics Series; 2015. p. 471–84.
